# Discovery of Goethe’s amber ant: its phylogenetic and evolutionary implications

**DOI:** 10.1038/s41598-026-36004-4

**Published:** 2026-01-22

**Authors:** Brendon E. Boudinot, Bernhard L. Bock, Daniel Tröger, Michael Weingardt, Jörg U. Hammel, Veit Grabe, Mónica M. Solórzano-Kraemer, Kenny Jandausch, Jill T. Oberski, Thomas Schmuck

**Affiliations:** 1 Senckenberg Gesellschaft für Naturforschung, Senckenberganlage 25, 60325 Frankfurt am Main, Germany; 2 Friedrich-Schiller-Universität Jena, Institute for Zoology and Evolutionary Research, Jena, Germany; 3 National Museum of Natural History, Smithsonian Institution, 10th & Constitution Ave. NW, Washington, DC USA; 4 Institute of Materials Physics, Helmholtz-Zentrum Hereon, Max-Planck-Straße 1, 21502 Geesthacht, Germany; 5 Max-Planck-Institute for Chemical Ecology, Hans-Knöll-Straße 8, 07745 Jena, Germany; 6 Universitätsklinikum Jena, Institute for Anatomy I., Teichgraben 7, 07743 Jena, Germany; 7 Klassik Stiftung Weimar, Goethe National Museum, Frauenplan 1, 99423 Weimar, Germany

**Keywords:** Ecology, Ecology, Evolution

## Abstract

Museum collections remain essential scientific resources, especially when revisited using modern analytical techniques. In an interdisciplinary study, we examined the overlooked amber collection of Johann Wolfgang von Goethe (1749–1832), polymath and pioneer of art and natural science. Using synchrotron-based micro-computed tomography (SR-µ-CT), we identified a fossil ant from Baltic amber (Eocene ~ 47–34 Ma) in Goethe’s collections. The specimen is assigned to †*Ctenobethylus goepperti* (Mayr in Die Ameisen des Baltischen Bernsteins. Beiträge zur Naturkunde Preussens, 1868), which we redescribe and re-diagnose, proposing †*Eldermyrmex exsectus* Dubovikoff et Dlussky, 2019 as its junior synonym (syn. nov., comb. nov.). We further infer a potential sister-group relationship with the extant genus *Liometopum* Mayr, 1861, suggesting that †*C. goepperti* may have been a dominant arboreal species in warm-temperate coniferous forests, a scenario which is supported by its abundance in Baltic amber. Critically, our results document endoskeletal structures in a Cenozoic fossil ant, underscoring both the morphological value of historical collections and the lasting scientific legacy of Goethe’s naturalist vision.

**Supplementary Information:**

The online version contains supplementary material available at 10.1038/s41598-026-36004-4.

## Introduction

Johann Wolfgang von Goethe (1749–1832), the polymathic poet, statesman, and naturalist, maintained a deep and lifelong engagement with the natural sciences, particularly geology, mineralogy, botany, and morphology. While he is most renowned for his literary works and philosophical contributions, Goethe also made original scientific observations, including his rediscovery of the *os intermaxillare* (premaxillary bone) in humans (Haas^1^, and c.f. Barteczko^2^), and played a key role in developing morphology as a scientific discipline. It has even been argued that Goethe’s artistic direction was of such a kind that it drove him necessarily to science (Steiner^3^). He conceptualized nature as a system in continuous transformation, driven by the interplay of form and function, a view that shaped his approach to classification and scientific observation. From this perspective of natural dynamism, he developed the concept of *Reihenbildung* (series formation) to describe orderly variation in natural systems (Mierbach^4^), a precursor to ideas central in evolutionary biology and systematics.

Goethe’s scientific practice was deeply intertwined with his personal collections, now housed in the Goethe-Nationalmuseum in Weimar (Thuringia, Germany). Unlike many historical collections, his geological and mineralogical specimens, comprising over 18,000 objects, have been preserved nearly in their entirety and remain in their original location. Many of the objects reside in the same boxes and cabinets Goethe used, bearing his handwritten labels. This continuity offers a unique opportunity to study Goethe’s scientific perspective through the material record.

Within these collections, amber occupies only a modest place. Classified not among fossils but among combustible substances (“*brennliche Wesen*”), Goethe catalogued amber under fossil resins (“*fossile Harze*”), reflecting late 18th-century systematic conventions that grouped it with substances like bitumen and jet. A catalog from 1978 (Prescher^5^) listed only four amber entries; however, recent re-inventorying revealed at least 40 pieces, including two samples of “amber earth” (“*Bernsteinerde*”). These are housed in their original cabinet (“Schrank II”) and include specimens from the Baltic region, including Gdańsk and Pomerania. Although Goethe himself never made explicit mention of biological inclusions, and any scientific study of amber bioinclusions started only near the end of his life, especially in Germany with Georg Carl Berendt’s “*Die Insekten im Bernstein*” (The insects in amber) in 1830, our examination revealed three insect inclusions within two pieces: two nematoceran flies and a worker ant.

Here, we present a detailed examination of this fossil ant, †*Ctenobethylus goepperti* (Mayr, 1868^6^), using synchrotron radiation-based micro-computed tomography (SR-µ-CT). The scan allowed for a three-dimensional reconstruction of the specimen, revealing anatomical features that were previously inaccessible, particularly the tentorium and prosternum, two endoskeletal structures never before documented in Cenozoic fossil ants. Our study provides a redescription and re- diagnosis of †*Ctenobethylus*, establishing †*C. goepperti* as a senior synonym of †*Eldermyrmex exsectus* Dubovikoff et Dlussky, 2019 (syn. et comb. nov.). The refined morphological assessment allows for improved systematic placement and paleoecological inference, evidencing an arboreal lifestyle in Eocene coniferous forests. Through this work we document paleontological discoveries and provide a rare synthesis of Enlightenment-era collecting and modern analytical science, illustrating the enduring scientific value of historical collections. It also invites reflection on Goethe’s holistic and integrative approach to knowledge, an approach grounded in careful observation, the ordering of nature, and the search for underlying principles that bridge form and function.

## Results

### Examined inclusions

**Fig. 1:**
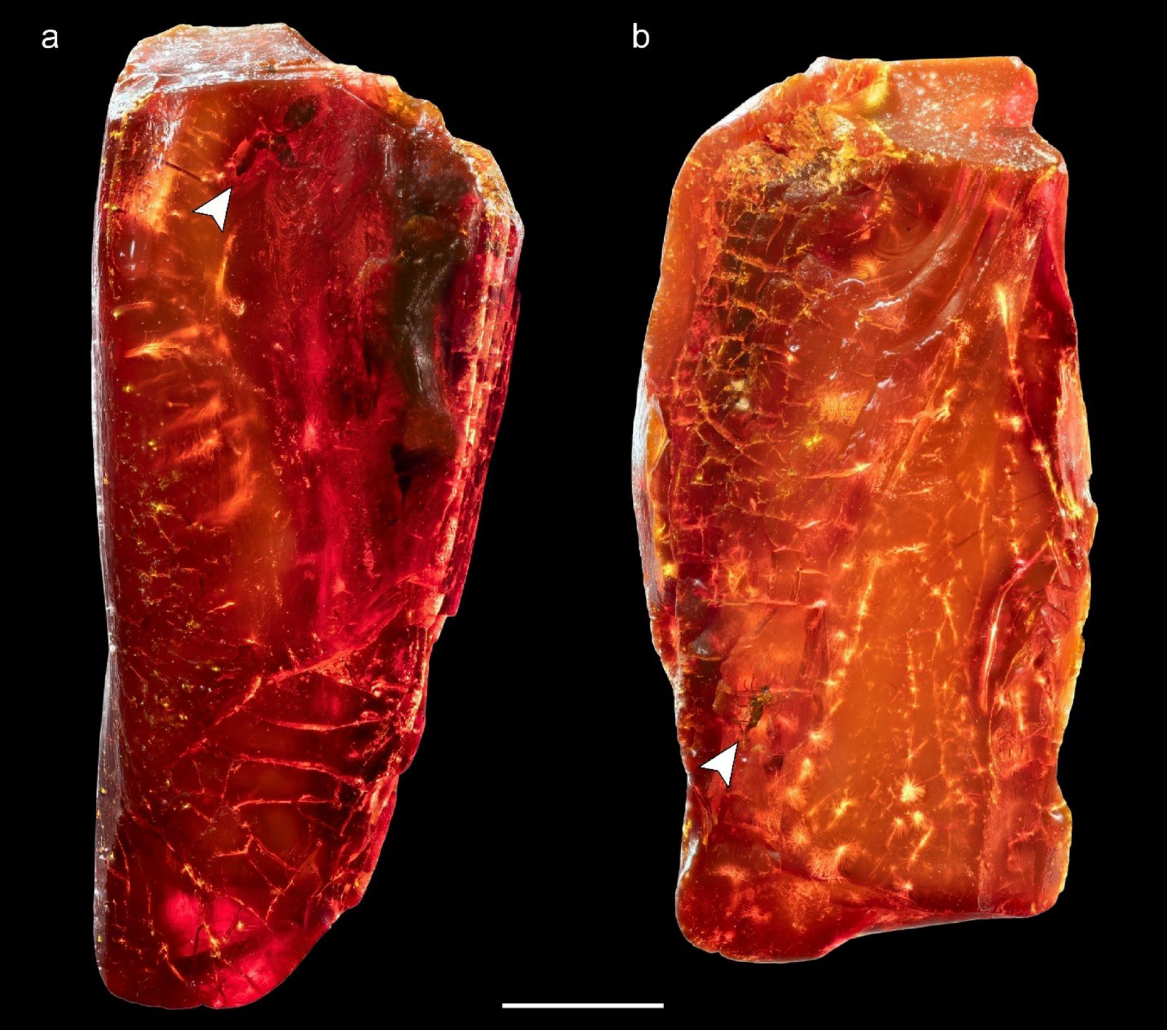
Amber piece 1552.b showing bioinclusions. Arrow in **a** (top): inclusion of †*Ctenobethylus goepperti*; arrow in **b** (bottom): inclusion of the Sciaridae. Scale bar 5 mm.

**Fig. 2:**
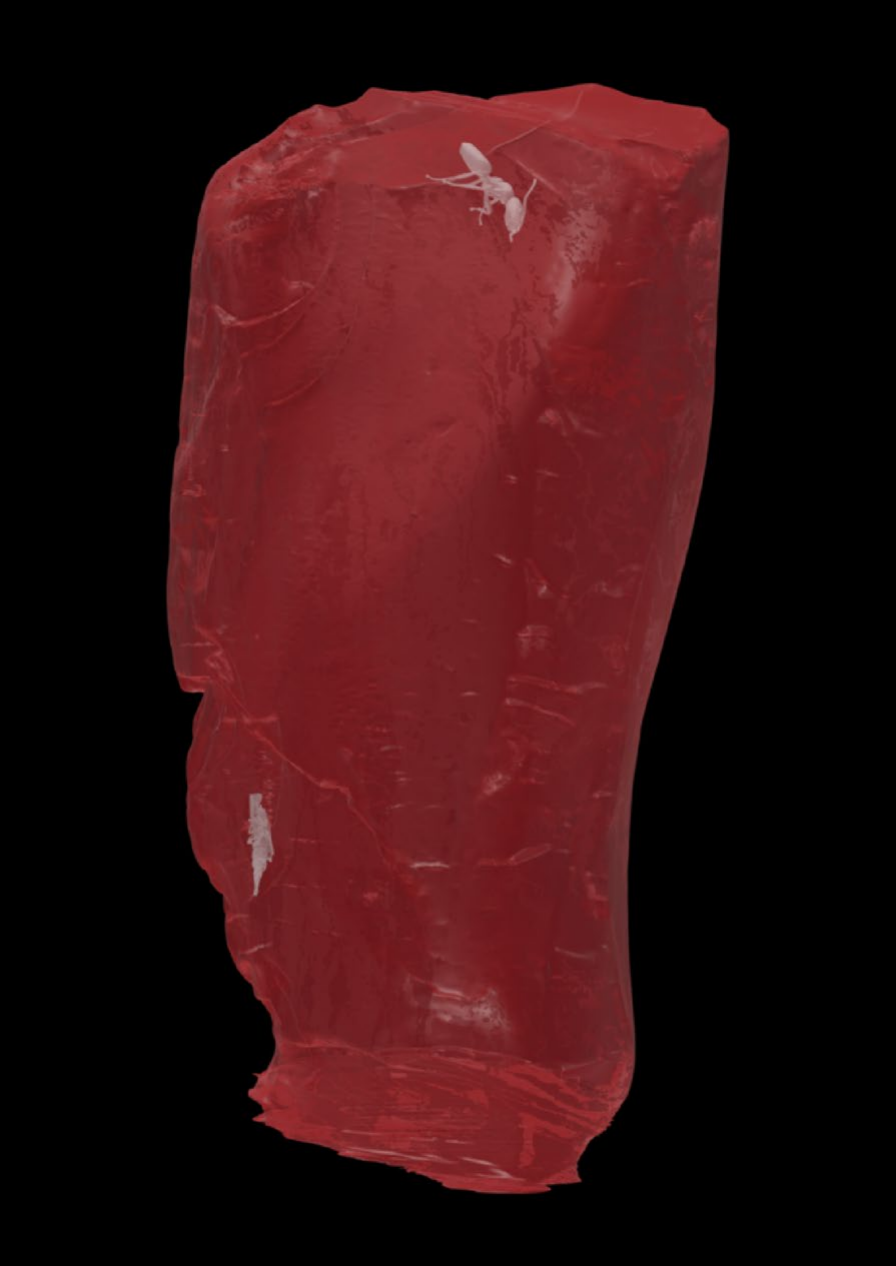
3D render of amber piece 1552.b with its corresponding bioinclusions in their position. Top: †*Ctenobethylus goepperti*. Arrow: sciarid gnat coated close to the rear surface of the backside of the amber piece in this view. 3D model available on Sketchfab: https://sketchfab.com/3d-models/goethe-amber-inclusions-d2d1f7f09a3e43e2a12f5467697cd3a2.

**Fig. 3:**
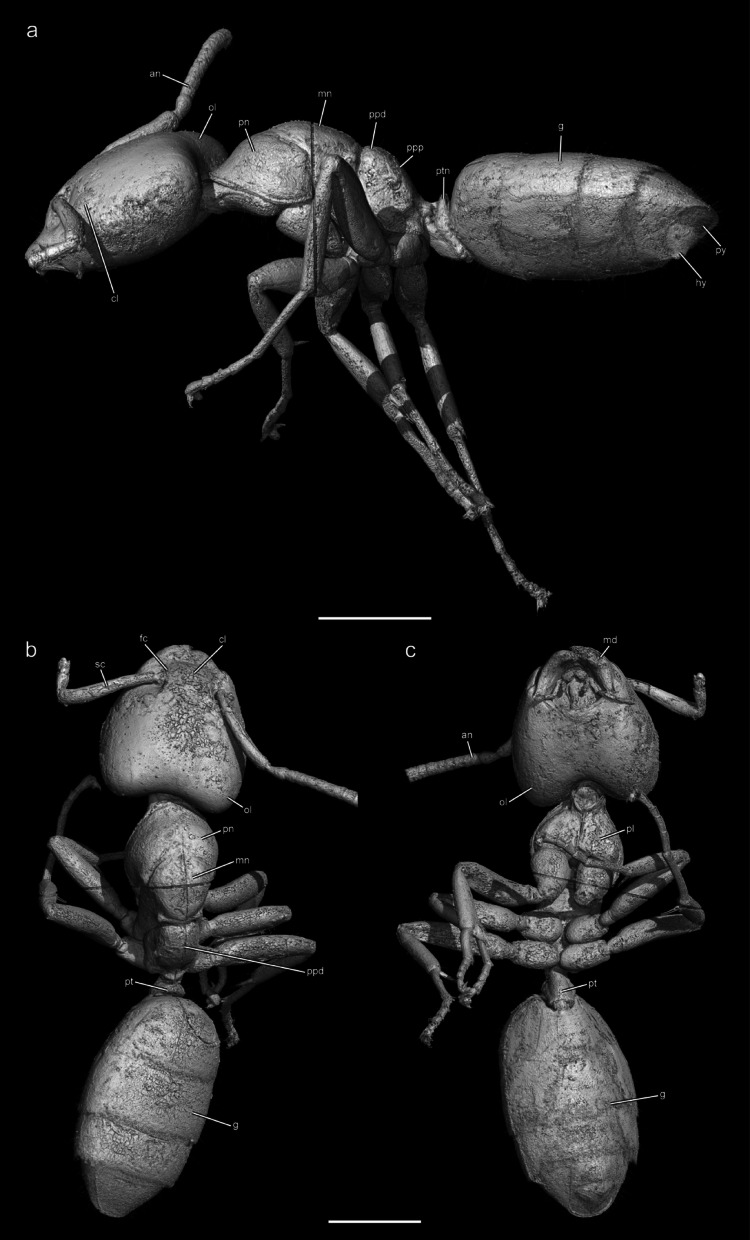
3D renders of †*Ctenobethylus goepperti* in amber piece 1552.b. **a** Lateral view. **b** Dorsal view. **c** Ventral view. Abbreviations: an = antenna; ce = compound eye; cl = clypeus; fc = frontal carina; g = gaster; hy = hypopygium; ol = occipital lobe; mn = mesonotum; pl = propleuron; pn = pronotum; ppd = propodeum, dorsal face; ppp = propodeum, posterior face; ptn = petiolar node; py = pygidium; sc = scape. Scale bars 0.5 mm. 3D model available on Sketchfab: https://sketchfab.com/3d-models/ctenobethylus-goepperti-adf1d4e9abd5416ca0c401ad6fa2caa1.

We examined all 40 amber pieces using a stereomicroscope. Most were opaque, a natural state of many Baltic amber pieces, and a sign of an ongoing aging process. Of the 40, we used synchrotron radiation to exploratorily scan 30 pieces, including the two pieces that contained the three insect inclusions. In one piece, labeled ‘ID 1552.b’, we found one ant that we identified as †*Ctenobethylus goepperti* (Hymenoptera: Formicidae) and a dark-winged fungus gnat (Diptera: Sciaridae) (Fig. 1, 2, 3 and Suppl. Fig. 3). The other piece, labeled ‘ID 1550.d’, contained a blackfly (Diptera: Simuliidae) (Suppl. Figs. 1 and 2). Finer identifications for the two Diptera were not achieved yet, as their state of preservation could pose an insurmountable obstacle, even with µ-CT rendering. The family identity of the two flies was confirmed via contact with an expert (pers. comm. Björn Rulik, March 12, 2023).

### Systematic paleontology

Order **Hymenoptera** Linnaeus, 1758.

Family **Formicidae** Latreille, 1809.

Subfamily **Dolichoderinae** Forel, 1878.

Tribe **Tapinomini** Emery, 1913.

†***Ctenobethylus Brues, 1939.***

 = †*Eldermyrmex* Dubovikoff et Dlussky, 2019 (syn. nov.)

Type species: †*C. succinalis* Brues, 1939 (= j. syn. of †*C. goepperti* (Mayr, 1869^6^): Brown, 1974^7^, p. 214).

*Diagnosis*: †*Ctenobethylus* is endemic to Eocene ambers (42–34 Ma) from the Baltic region (Baltic, Bitterfeld, Danish-Scandinavian, Rovno, Belarus; Dlussky and Rasnitsyn^8^; Dlussky and Perkovsky^9^) and uniquely identifiable among all Dolichoderinae extant and extinct by the following combination of morphological conditions: (1) head cordate, with large occipital lobes; (2) compound eyes situated in the anterior half of the head; (3) frontal carinae sharp; (4) frontal carinae wideset, being separated by more than one of their own lengths; (5) anterior clypeal margin linear; (6) scapes short, not exceeding the posterior head margin; (7) mesosoma compact; (8) mesonotum well-developed and long; (9) propodeal dorsal surface distinctly shorter than the posterior surface in lateral view; (10) petiole with a distinct, thin node; and (11) petiole without pronounced posterior elongation (see Fig. 3a–c). The specific differentiation of †*Ctenobethylus* relative to other Dolichoderinae is addressed in the Discussion under “Systematic implications” below. For a complete description and rationale for synonymization of †*Eldermyrmex*, see the Supplementary Results.

## Discussion

*Systematic implications*: Placing †*Ctenobethylus* among Dolichoderinae is not trivial. We accept the placement of the genus in Tapinomini, first proposed by Ward et al.^10^, based on a process of elimination, wherein we used the favored multi-gene topology of Ward et al.^10^ for detecting morphological consistencies across the subfamilial phylogeny. Our reasoning for each dolichoderine tribe follows below.

*Tribe Bothriomyrmecini.* Although *Arnoldius*, *Bothriomyrmex*, and *Chronoxenus* have largely similar mesosomal forms (mesosoma compact, dorsal propodeal face short), †*Ctenobethylus* cannot be placed in Bothriomyrmecini, as it lacks the synapomorphic facial modifications of the constituent genera of this tribe. Specifically, the posterior clypeal margin of †*Ctenobethylus* extends posteriorly between the antennal toruli (vs. synapomorphy: this margin ending at around the anterior torular margin in all bothriomyrmecine genera), the antennal toruli are wideset, separated by more than one of their own lengths (vs. closely approximated in *Loweriella* and *Ravavy*), the frontal carinae are well-defined (vs. synapomorphy: effaced in *Arnoldius*, *Bothriomyrmex*, and *Chronoxenus*), and the medial hypostomal lamella is well-developed (vs. synapomorphy: reduced or absent in all bothriomyrmecine genera).

*Tribe Leptomyrmecini.* The propodeum of †*Ctenobethylus* does not appear diagnostic at first examination but is in fact distinct in comparison to the Leptomyrmecini. Across the leptomyrmecines, the dorsal propodeal surface is distinctly developed, being longer than the posterior surface (*Iridomyrmex*, *Ochetellus*, *Papyrius*, *Philidris*, *Turneria*, *Axinidris*, *Forelius*, *Anillidris*), about as long as the posterior surface (*Azteca*, *Linepithema*, *Anonychomyrma*, *Dorymyrmex*), or distinct but shorter than the posterior surface (*Nebothriomyrmex*). *Doleromyrma* and various *Leptomyrmex* have short dorsal propodeal surfaces but differ from †*Ctenobethylus* as the former genus has frontal carinae that are close-set, and the latter has an elongate body form and lacks the medial hypostomal lamella. Certain leptomyrmecine genera are further distinguished through petiolar form (high-squamate in *Iridomyrmex*, *Ochetellus*, *Papyrius*), eye form (anteriorly converging in *Turneria*), and almost all leptomyrmecines have anterior clypeal margins that are convex or sinuate in some manner, with the most limited exceptions being *Dorymyrmex* and *Anillidris*, which have weakly convex margins but are otherwise distinguished by several other conditions (e.g., psammophore development, torular separation).

*Tribe Dolichoderini.* The highly derived Dolichoderini can be excluded, as †*Ctenobethylus* lacks the flange-like anterolateral hypostomal teeth, a strong, large petiolar node, and the thick cuticular sculpture. Thus, only the tribe Tapinomini remains, as apparently inferred by Ward et al.^10^, Shattuck^11^^,^^12^, and Bolton^13^^,^^14^).

*Tribe Tapinomini.* Within the Tapinomini, †*Ctenobethylus* has been previously synonymized with the extant, arboreal genus *Liometopum* (Shattuck^15^), an inference that was not explained, or otherwise considered in detail (Dlussky and Perkovsky^9^). Given that the petiole of †*Ctenobethylus* plesiomorphically retains a small but distinct petiolar node and short posterior petiolar tube, it is reasonable to exclude the genus from the *Axinidris* + *Technomyrmex* clade and from the *Tapinoma* crown clade. *Axinidris* and *Technomyrmex*, further, have comparatively long to definitely long dorsal propodeal surfaces. Among *Liometopum* and the remaining tapinomine genera, *Aptinoma* and *Ecphorella*, †*Ctenobethylus* clearly differs from *Ecphorella* in having a linear anterior clypeal margin. Fascinatingly, †*Ctenobethylus* is morphologically intermediate between *Aptinoma* and *Liometopum*, and the relationship of these two taxa is in a virtual polytomy with *Tapinoma* given the analyses of Ward et al.^10^ and the reanalysis of Boudinot et al.^16^.

The available morphological evidence suggests that †*Ctenobethylus* is a stem group of *Liometopum*: (1) the second submarginal cell is present (see Fig. 45 of plate III in Mayr^6^ and the venational mapping of Boudinot et al.^16^ in Fig. 5; vs. absent in *Aptinoma*; note that reduction of this cell is prone to homoplasy across all dolichoderine tribes); (2) the head is distinctly cordate (vs. very weakly lobate posteriorly in *Aptinoma*); (3) the teeth are relatively coarse (vs. fine in *Aptinoma*); (4) the petiolar node is distinctly developed, raised above the levator process, and anteroposteriorly narrow (vs. node not distinct, not raised above process, broad in *Aptinoma*); (5) antennomere III is about half the length of the pedicel (vs. about as long as the pedicel in *Liometopum*); (6) the scape is short, not exceeding the posterior head margin in repose (vs. scape long as specified in *Liometopum*); and (7) the body lacks elongate erect setae (vs. with such setae in *Liometopum*). The mesosoma of †*Ctenobethylus* is further similar to *Liometopum* in having a large, cavernous metapleural gland opening and long mesonotum. Notably, the mesonotum of ant workers does not receive musculature; thus, this is primarily a structural feature that influences the geometry of the pronotum, and by consequence, the head. Development of the metanotum and impression of the metanotal groove does not appear to have much bearing on the distinction of †*Ctenobethylus* relative to *Liometopum* and *Aptinoma*.

### Paleoecological implications

†*Ctenobethylus goepperti* is the most frequently encountered species of ants in Baltic amber, yet its abundance has paradoxically contributed to its scientific marginalization. However, through our renewed morphological considerations, particularly the probable sistergroup relationship to *Liometopum*, it becomes possible to make inferences of the biology of this fossil species. In terms of abundance, it is worth re-examining specimens of †*Liometopum oligocenicum* to determine whether they do, in fact, belong to *Liometopum*. Regardless, extant *Liometopum* are arboreal carton-nesters and form massive colonies that may extend from tree to tree. It is therefore possible that †*C. goepperti* was a dominant arboreal species in the humid and warm-temperate coniferous forests of Eocene Europe (e.g., Sadowski et al.^17^), which went extinct during the major climatological changes leading to the present day. Dlussky and Perkovsky^9^ previously speculated that the robust mandibles of †*C. goepperti* may have been useful for an arboreal lifestyle, specifically for excavating or boring wood. Where *Liometopum* are absent in contemporary central Europe, possibly due to Pleistocene glaciation, they are replaced by *Lasius* in the *niger* clade, by species of both the *brunneus* and *niger* groups as well as the highly derived *fuliginosus* group. In this light, turnover of the temperate Eocene ant fauna in European is further evidenced by the high abundance of extinct *Formica* species and †*Lasius schiefferdeckeri* in Baltic ambers and the very young age of crown *Lasius* (Boudinot et al.^18^). Seemingly a cursed genus given its taxonomic history (e.g., Shattuck^15^), †*Ctenobethylus* now appears to be a paleoecologically important taxon, being the likely Eocene analog of *Liometopum*.

### Historiographical reflections

Goethe’s legacy in the natural sciences extends far beyond his renowned *Farbenlehre* (Theory of Color; Goethe^19^) and provides the poetic impetus for the journal *Nature* (Huxley^20^; see Suppl. Text Box), but also encompasses sustained engagement with geology, mineralogy, and biological form. Best known among scientists for his rediscovery of the human premaxilla and his formulation of the *Urpflanze* or archetypal form in plant morphology, Goethe helped lay the groundwork for comparative morphology and the study of metamorphosis in biological systems. Through both theory and practice, Goethe emphasized perceptual and conceptual synthesis, an aesthetic empiricism that sought truth through close observation, iterative experimentation, and attention to form and transformation, *i.e.*, Goethe’s *Naturphilosophie*.

Goethe shaped our modern patterns of biological inquiry by being among the first to integrate ideas about structure, differential development, and process over time. In particular, he distilled organismal science into the living concepts of morphology and metamorphosis, together, a broadly applicable theoretical framework for the consideration of analogy and homology (Hall^21^, Levit et al.^22^). Distilling complex reality into perceptible patterns once earned him a commendation as “the Copernicus and Kepler of the organic world” (Steiner^23^), and in order to shape this mentality, he placed great emphasis on the relationship between the investigator and their object of study. Namely, comparative morphology extends beyond simple comparison of anatomical facts; it requires direct human perspective to reveal the underlying metamorphosis and broader significance. From a Goethean perspective, comparative morphology is not only descriptive but also explanatory, even complementing modern molecular approaches in evolutionary developmental biology (Toni^24^).

Across multiple fields of study, it becomes clear that Goethe wanted to understand time and process, not only pattern (Sullivan^25^). In botany, Goethe sought the form of the *Urpflanze* that is contained in each plant today and how it manifests as variable leaf phenotypes (i.e., its processes of development) (Goethe^26^). He also endeavored to draw similar conclusions about the geological world, writing at length about granite and its status as the oldest of Earth’s ancient stones. The incredible antiquity of our planet, a long history documented in text-like layers in stones and mountains, was at the time a revolutionary idea with both religious and scientific implications. Geology was just beginning to develop from a classificatory scheme into a field concerned with the Earth’s formation and evolution (Sullivan^25^). In this context Goethe’s geological collections are herald of the shifting scientific perspectives of the era. Furthermore, reminiscent of modern museum archives, Goethe believed collections such as the *en vogue* “cabinets of curiosities” were shared intellectual capital rather than personal property. In the collection-themed novella “*Der Sammler und die Seinigen*”, co-written with Friedrich Schiller (1759–1805), he highlights the interpersonal aspects of collecting, such as corresponding about a collection’s inheritance and comparing and transferring specimens across different collections, making clear that these information-rich assemblages transcend a single owner (Schellenberg^27^).

Amber was not central to Goethe’s scientific output, yet it occupied an intriguing intersection of his interests. Classified in Schrank II of his mineral system under combustible substances of economic value (“*Brände*”), amber was grouped with resins and other organic materials rather than with fossils. His amber holdings, some 42 catalogued pieces (of which 40 could be found) today preserved by the Klassik Stiftung Weimar, were acquired primarily from contemporary dealers, or gifts from friends with synergetic interests, such as Johann Georg Lenz (1748–1832) in 1796. Goethe was familiar with the fact, that amber could contain biological inclusions, as he held a friendship with Friedrich W. H. von Trebra (1740–1819), corresponded with him, read his works, quoted it, and kept two copies of his “*Mineraliencabinett gesammlet und beschrieben*” in his library that states on p. 125 “*[…] das Stück Bernstein […] welches ein Lorbeerblatt, und ein Paar kleine Insecten darauf einschließt*” (Trebra^28^). Thus, while he was evidently aware of the paleontological potential of amber, he did not examine these for inclusions, as suggested by his omission of contemporary works like Sendel^29^ and his lack of commentary on biological inclusions. Rather, Goethe conducted optical experiments using amber, attempting to observe visual effects like entoptic phenomena. These efforts culminated in the “*Entoptische Farben*” chapter of his 1820 supplement to *Farbenlehre*, demonstrating amber’s experimental utility in Goethe’s empirical investigations of light and vision.

In pursuit of botanical, geological, and optical knowledge, Goethe shared energizing conversations with famed naturalist and explorer Alexander von Humboldt (1769–1859), who was likewise trying to understand nature through both empirical analysis and aesthetic perception. These exchanges fueled Goethe’s desire to collect and curate his personal collections, creating a continuous cycle of artistic and scientific pursuits. Even today, aesthetic considerations remain significant to scientists. In a 2021 survey of 3442 scientists, 91% reported they find it important to encounter beauty, awe, and wonder in their work; moreover, 94% believed that science helps us better access the beauty that actually exists in the world (Jacobi et al.^30^). More frequent experiences of wonder and awe also correlate with higher job satisfaction and better mental health.

**Fig. 4:**
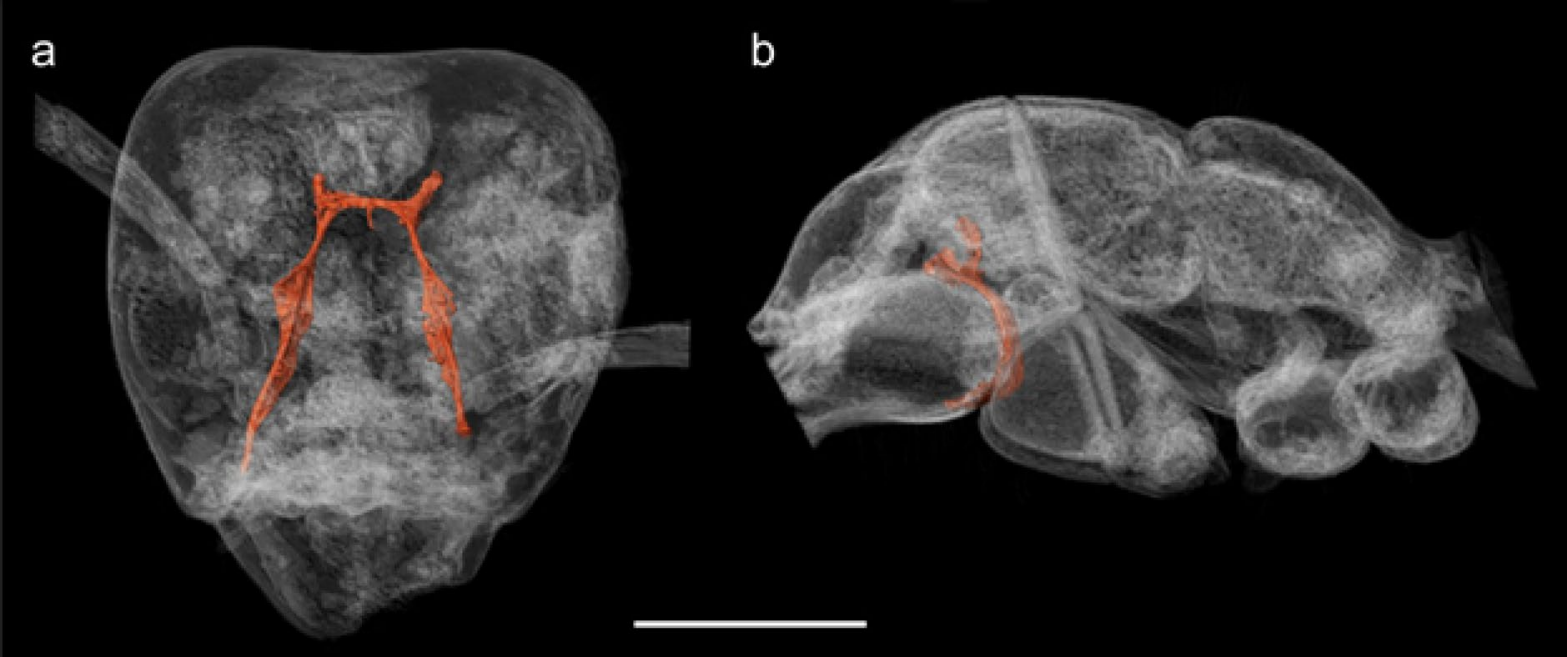
Transparent 3D renders of †*Ctenobethylus goepperti* in amber piece 1552.b. **a** Head frontal view showing the tentorium. **b** Mesosoma seen from lateral showing the profurca, legs removed. Scale bar 0.5 mm. 3D model available on Sketchfab: https://sketchfab.com/3d-models/ctenobethylus-goepperti-f816f0a528e540a8959fa96361c395b8.

Fittingly our investigation bridges Enlightenment-era inquiry and modern morphological bioinformatics, or phenomics (i.e., big-data morphology; Deans et al.^31^). Using synchrotron radiation-based micro-computed tomography (SR-µ-CT), we scanned Goethe’s amber collection for bioinclusions. Remarkably, only two of the 40 pieces contained visible fossil insects: two nematoceran flies and an ant, †*Ctenobethylus goepperti*. This study presents the first high-resolution reconstruction of the ant (Fig. 3), permitting redescription of the species and genus, documentation of internal structures like the tentorium and prosternum (Fig. 4a and b), and a new taxonomic synonymy. These findings reanimate a neglected piece of Goethe’s collections and exemplify the growing potential of historical collections for generating new scientific knowledge.

The term “collectomics”, recently advanced by Sigwart et al.^32^, provides an apt framing for the epistemological and practical value of this work. Collectomics treats natural history collections not merely as repositories of objects, but as structured, information-rich datasets subject to modern scientific scrutiny. Our study contributes to this emerging framework by applying advanced imaging technologies to Goethe’s archival specimens, without altering their cultural integrity, and demonstrating that even long-frozen collections can yield unexpected scientific dividends. Ongoing imaging and provenance research work will improve the accessibility of these collections for cultural and scientific research (Schmuck^33^). This case also reflects Goethe’s own philosophy: that truth lies in the persistent, perceptive encounter with nature, and that empirical inquiry must be grounded in both historical consciousness and aesthetic sensibility.

As Goethe once wrote:

“*Weder Fabel noch Geschichte, weder Lehre noch Meinung halte ihn ab zu schauen*. ”

(May neither legend nor history, neither theory nor opinion, prevent him from seeing.)

– J.W. von Goethe, letter to the Duke of Saxony-Gotha, 1780 (as per Safranski and Dollenmayer^34^)

## Conclusion

**Fig. 5:**
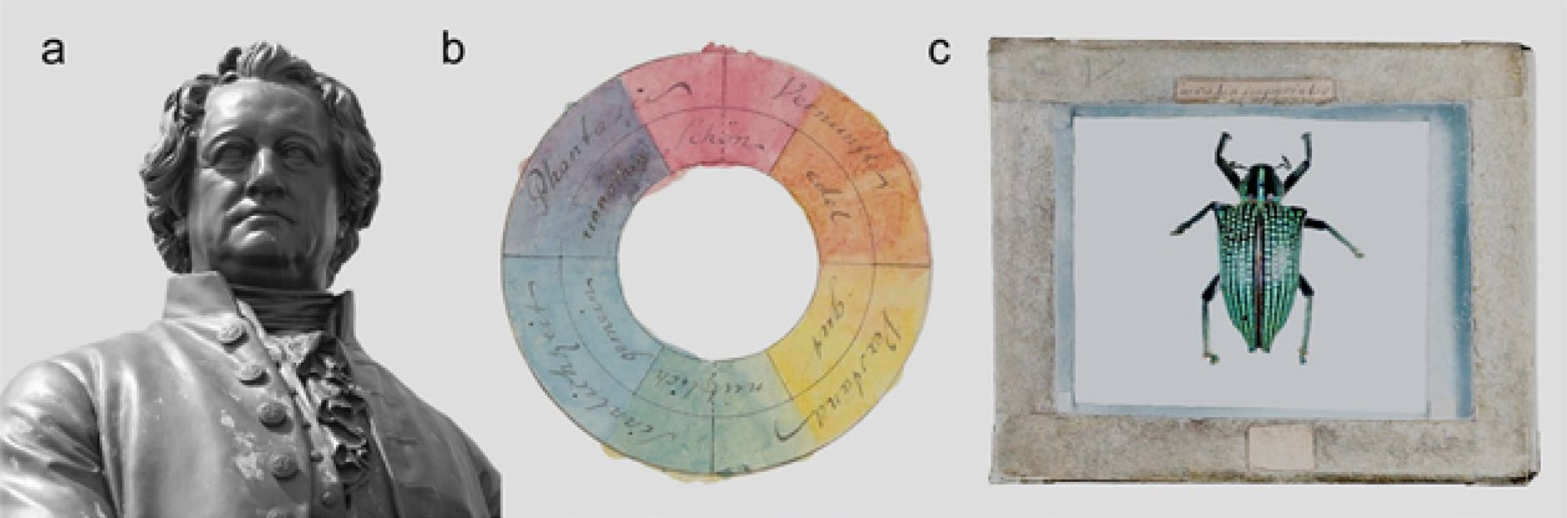
Goethe as a Natural philosopher. **a** Goethe and Schiller Monument in Weimar. **b** For Goethe, art, science, and humanity were inextricably linked. He was particularly invested in color theory and prisms on both a physical and a philosophical level; he painted and annotated this color wheel in 1809^35^ for his Farbenlehre to symbolize the “human spirit and soul life” (image taken from: Freies Deutsches Hochstift, Frankfurter Goethe Museum; https://goethehaus.museum-digital.de/singleimage?imagenr=35554). **c** Goethe collected many natural objects; this specimen is the last remaining insect in Goethe’s collections (without the amber inclusions). The label states “*Curculio imperialis*”, today known as *Entimus imperialis* (Forster J.R, 1771), an endemic beetle species from Brazil.

Amber can preserve biological structure at unparalleled fidelity, and the Goethe collection, preserved through cultural significance rather than scientific design, has now yielded new insights through modern imaging. Our phenomic redefinition of †*Ctenobethylus goepperti* demonstrates the continued potential and relevance of historical collections for systematic revision and paleobiological interpretation. Through this lens, Goethe’s own epistemological commitment to observation, metamorphosis, and morphological synthesis finds renewed relevance. This study reflects the intersecting values of historical inquiry and contemporary science: where morphology joins informatics, where amber infirms evolutionary biology, and museum collections continue to generate knowledge when examined with care and curiosity, whether these are geological, biological, or cultural (Fig. 5). In a poetic inversion, we now use advanced imaging tools to peer through the same amber Goethe once used to explore vision. While Goethe opposed artificial enhancements of perception in principle, he embraced tools like microscopes and prisms when they aided genuine observation. This study honors that spirit by integrating visual technology with empirical inquiry and highlights the enduring value of historical collections for modern science.

## Materials & methods

### Materials

We examined a total of 40 amber pieces found in Goethe’s collections held in Weimar (Germany). Of these 40 pieces four are presumably ambergris (produced in the digestive system of sperm whales). The remaining 36 pieces were classified as Baltic amber (47–34 Ma) (Kosmowska et al.^36^, Kasiński et al.^37^). Six of these were not scanned, as we could already observe under the microscope that they did not hold any bioinclusions. In total 30 pieces were suitable for scanning.

### Photography

No piece was ground or polished, as the objects are of significant cultural value. Pieces were cleaned with a dust blower and Super Soft Dusting Brush (Preservation Equipment Ltd. Vinces Road, Diss, Norfolk, UK). Stacks of partially focused images were taken of the amber pieces with a Canon EOS R5 equipped with Canon EF 100 mm f/2.8L Macro IS USM (Canon, Krefeld, Germany). For focus stacking, the internal camera software was used. The camera was mounted on a Kaiser copy stand (Kaiser Fototechnik GmbH & Co. KG, Buchen, Germany). The scene was illuminated with a Euromex LE.5211–230 cold light source for stereomicroscopy (Euromex, Papenkamp, the Netherlands) equipped with three gooseneck lamps to adjust light conditions and prevent reflections. Underneath the camera a blurred glass plate was positioned over a black sprayed Kapa® box (Kohlschein GmbH & Co. KG, Viersen, Germany). The amber pieces were placed on the glass plate in a petri dish filled with distilled water (Sadowski et al.^38^). Additional photographs were taken with the same camera and lens.

### Microtomography (µ-CT)

In total 30 pieces of Baltic amber from Goethe’s collections were first scanned at the Deutsches-Elektronen-Synchrotron (DESY), Hamburg, Germany and subsequently at the Max Planck Institute for Chemical Ecology, Jena, Germany.

### Synchrotron-radiation µ-CT: DESY

In two pieces animal inclusions were found (Fig. 1 and Suppl. Fig. 1) under the light microscope, these were scanned at the DESY using synchrotron radiation (SR-µ-CT) at the Imaging Beamline p05 (IBL) operated by the Helmholtz-Zentrum Hereon at the storage ring PETRA III. A photon energy of 18 keV and a sample to detector distance of 30–50 mm was used. Projections were recorded using a 50 MP CMOS camera system with an effective pixel size of 0.46 µm. 4001 projections were recorded for each tomographic scan at equal intervals between 0 and π, with an exposure time of 350 ms. When specimens were too large to fit into the field of view in the z-axis, we scanned overlapping sections and subsequently stitched them together. Tomographic reconstruction was done by applying a transport of intensity phase retrieval and using the filtered back projection algorithm (FBP) implemented in a custom reconstruction pipeline (Moosmann et al.^39^) using MATLAB (Math-Works) and the Astra Toolbox (Palenstijn et al.^40^, van Aarle et al.^41^^,^^42^). For further processing, raw projections were binned two times resulting in an effective pixel size of the reconstructed volume of 0.913 µm. For segmentation and visualization, the 32-bit .tif image sequences were converted to 8-bit files and down sampled twofold with Fiji (Schindelin et al.^43^), resulting in an effective pixel size of 1.826 µm. Pieces with no visible bioinclusions were “pre-scanned” and checked if there are any specimens inside (see Suppl. Tables 1 and 2).

### Conventional µ-CT: MPI

All ambers pieces were pre-scanned with a SkyScan 1272 (Bruker, Belgium) equipped with an Xray sensitive xiRay16 (4872 × 3248) (Suppl. Table 2). One of the seven pieces labeled with ID 1552.b containing †*Ctenobethylus goepperti* and a sciarid fly was irradiated with a voltage of 25 kV and a current of 125 µA. The exposure time for one image was 5.19 s. A pixel size of 7.4 × 7.4 µm^2^ was achieved. The 360° scan was accomplished in 0.2° steps. Three additional pieces, suspected of containing bioinclusions, labeled with ID 1550.d were quick scanned with the same parameters, except for source voltage of 23, 27 and 30 kV and a current of 130 µA.

### Data segmentation and rendering

µCT-image stacks were segmented and 3D-reconstructed using Amira 6.0.1 (Thermo Fisher Scientific, Waltham, Massachusetts, USA) and Dragonfly 2022.1 (Object Research Systems, Montreal, Quebec, Canada). tif image stacks and files containing the isosurfaces were exported with the Amira macro “Multi-Export” (Engelkes et al.^44^). The isosurfaces were reduced and smoothed under the following parameters: ItTotal: 5; smooth: iteration: 4, lambda: 0.6; reduction: 0.7. The resulting .tif image stacks were volume rendered in VGStudio Max 2.0 (Volume Graphics, Heidelberg, Germany) using the option Phong reflection model. The isosurfaces were further smoothed (modifier: smooth and option shade smooth) with Blender 3.2.0 (Blender Foundation). The 3D-Models were uploaded to the 3D model repository Sketchfab (https://sketchfab.com) using the free blender plugin: Sketchfab for blender 1.5.0 (https://github.com/sketchfab/blender-plugin/releases/tag/1.5.0).

### Image plates

Image plates were compiled using Adobe Photoshop (v. 24.1.0) (Adobe, San Jose, USA). Lettering was added with Adobe Illustrator (v. 27.2).

### Authenticity verification

There is no doubt the material is any other than Baltic amber, an assumption confirmed by spectroscopy for amber piece 1552.b. Since these amber pieces are highly brittle as a result of their basic storage conditions in the hallway to Goethe’s “Arbeitszimmer” (study room), a small piece unintentionally broke off of specimen 1552.b, the piece that contains two bioinclusions, during transportation and scan preparations. The crumb was sent to the International Amber Association (IAA; Gdańsk, Poland) for UV fluorescence and Fourier-transformed infrared spectroscopy (FT-IR). The results showed that the piece is true Baltic amber (Report No 41992_17022023). Due to their inestimable cultural value, we refrained from examining further pieces. As this would mean sending the whole amber pieces to the IAA or cutting off small flakes, that would bear the risk of harming the objects.

### Morphological comparisons

Renders of the synchrotron-scanned specimen were used to evaluate gross morphology as well as structures that are otherwise hidden or internal; the specimen could not be clearly seen under standard light microscopic conditions. As needed to evaluate given characters, comparisons were made to images hosted on AntWeb^45^ and the literature, particularly Richter et al.^46^^,^^47^^,^^48^^,^^49^^,^^50^ and Boudinot et al.^51^ for the tentorium. For the prosternum, we referred to Aibekova et al.^52^ and Aibekova et al.^53^. Terminology used for description and discussion follows these references for the head and mesosoma, with the addition of Lieberman et al.^54^ for the metasoma. Detailed comparisons of worker morphology were made for the following genera based on AntWeb (numbers in square brackets indicate taxon sampling within the indicated tribe): Bothriomyrmecini [5/5] (*Arnoldius*, *Bothriomyrmex*, *Chronoxenus*, *Loweriella*, *Ravavy*); Dolichoderini [1/1] (*Dolichoderus*); Leptomyrmecini [16/16] (*Anillidris*, *Anonychomyrma*, *Azteca*, *Doleromyrma*, *Dorymyrmex*, *Forelius*, *Froggattella*, *Gracilidris*, *Iridomyrmex*, *Leptomyrmex*, *Linepithema*, *Nebothriomyrmex*, *Ochetellus*, *Papyrius*, *Philidris*, *Turneria*); Tapinomini [6/6] (*Aptinoma*, *Axinidris*, *Ecphorella*, *Liometopum*, *Tapinoma*, *Technomyrmex*).

### Morphometrics

Measurements were taken from the 2D orthographic renders in parallel perspective of the fossil in full-face and lateral view using Adobe Illustrator. These data were recorded in pixels and translated into metric units, with results output in mm to the second decimal place. Abbreviations for the morphometrics are as follows.

*Measurements:*

A2L: *Pedicel (antennomere 2) length*. (Full-face view.) The length of the pedicel from its proximal inflection to its apical margin. Note: This and the next measurement are foreshortened in the present metrics.

A3L: *Antennomere 3 length*. (Full-face view.) The length of the third antennomere from its base to its apical margin.

CLL: *Clypeus length*. (Full-face view.) The length of the clypeus from the anteromedian point to the posteromedial-most point along the head midline.

EL: *Eye length*. (Lateral view.) The maximum length of the compound eye.

EW: *Eye width*. (Lateral view.) The width of the compound eye measured orthogonal to EL.

FCS: *Frontal carina spacing*. (Full-face view.) The minimum distance between the frontal carinae.

HL1: *Head length 1*. (Full-face view.) The midline length of the head from the anteromedian point of the clypeus to the posteromedian point of the posterior margin of the head, excluding the occipital lobes.

HL2: *Head length 2*. (Full-face view.) The midline length of the head from the anteromedian point of the clypeus to a digital line between the apices of the occipital lobes.

HLA: *Anterior head length*. (Full-face view.) The distance from the anteromedian point of the clypeus to a digital line drawn between the anteriormost points of the compound eyes.

HLP: *Posterior head length*. (Full-face view.) The distance from the posteriormost point of the compound eye to a digital line drawn between the posteriormost apices of the occipital lobes.

IOC: *Interocular distance*. (Full-face view.) The minimum distance between the compound eyes.

LOC: *Lateral ocular distance*. (Full-face view.) The minimum distance between the compound eye and the lateral margin of the head.

ML: *Mesosoma length*. (Lateral view.) The distance from the inflection point of the pronotum between its anterior and posterior portions and the posteriormost point of the metapleural region.

PL: *Petiole length*. (Lateral view.) The distance from the anterior base of the petiolar node (excluding the petiolar presclerites) to the posteriormost point of the petiolar tergum.

SL: *Scape length*. (Full-face view.) The maximum length of the scape excluding the bulbus and bulbus neck, averaged between the left and right antennae.

SPD: *Propodeal spiracle diameter*. (Lateral view.) The maximum diameter of the propodeal spiracle.

*Indices:*

AI: *Antennomeres 2, 3 index*. A3L/A2L*100.

CI: *Cephalic index*. HW/HL1*100.

CS: *Cephalic size*. (HW + HL1)/2.

EI: *Eye index*. EL/EW*100.

ES: *Eye scale*. EL/HW*100.

OI: *Occipital index*. HL1/HL2*100.

SI: *Scape index*. SL/HW*100. 508.

## Supplementary Information

Below is the link to the electronic supplementary material.

Supplementary Material 1

## Data Availability

All four scans are available at: http://www.morphosource.org/projects/000760923?locale=en
