# Supplementary material for: Discovery of Goethe’s amber ant: its phylogenetic and evolutionary implications
Source: Sci Rep. 2026 Jan 22;16:2880. doi: 10.1038/s41598-026-36004-4 (PMC12830952; doi:10.1038/s41598-026-36004-4)
Supplement: Supplementary file 1 — Supplementary Material 1 [file 41598_2026_36004_MOESM1_ESM.docx]

***Supplementary file 1***

***Supplementary methods***

**Table 1 |** **Amber pieces from Goethe’s collections with generated datasets scanned at**

**DESY.**

| **Prescher Nr.** | **Name** | **Quantity / scanned** | **Collection-ID**  **(SemperNr.)** | **Comment** | **DESY-Scan ID** |
| --- | --- | --- | --- | --- | --- |
| 1550.d | Bernstein von der Ostsee | 7/3 | S II 3111 | With empty  form label | fsu_155_1550d_SII_31_II  (Simuliidae) |
| 1552.b | Bernstein aus  Danzig | 7/2 | S II 3119 |  | fsu_154_1550.b, SII_31_19 (†*Ctenobethylus goepperti*) fsu_155_1550b_SII_31_19  (Sciaridae) |

With all remaining 25 pieces “pre-scans” were performed to check for additional relevant

bioinclusions, but no datasets were processed. All these pieces were again scanned at the MPI for Chemical Ecology (see Suppl. Table 2).

**Table 2.** **Amber pieces from Goethe’s collection scanned at the MPI for Chemical Ecology**.

| **Presch er-Nr.** | **Name** |  | **Quantity/ scanned** | **Collection-ID (Semper-Nr.)** | **Comment** | **DESY-Scan** |
| --- | --- | --- | --- | --- | --- | --- |
| 1550.a | Bernstein der Ostee | von | 2/2 | S II 318 |  | One piece with debris |
| 1550.b | Bernstein Danzig | aus | 2/2 | S II 319 |  | No inclusions |
| 1550.c | Bernstein der Ostsee | von | 3/3 | S II 3110 | With empty form label | No inclusions |
| 1550.d | Bernstein der Ostsee | von | 7/4 | S II 3111 | With empty form label | Three with debris |
| 1550.e | Bernstein der Ostsee | von | 4/4 | S II 3112 | With empty form label | No inclusions |
| 1550.g | Bernstein der Ostsee | von | 2/2 | S II 3114 | With empty form label | No inclusions |
| 1550.h | Bernstein der Ostsee | von | 1/1 | S II 3115 |  | No inclusions |
| 1551 | Bernstein aus der Gegend von Symbom | | 1/1 | S II 3117 | With empty form label | Lot of debris |
| 1552.a | Bernstein aus Danzig | | 1/1 | S II 3118 |  | No inclusions |
| 1552.b | Bernstein aus Danzig | | 7/4+1 | S II 3119 |  | No inclusions, one piece is a pearl, which was not scanned  /†*Ctenobethylus goepperti* and  Sciaridae |

Not all amber pieces of Goethe’s collection have been scanned, as some of them did not contain inclusions, which could be observed under the stereomicroscope. Others weren’t suitable for scanning, such as the *Honigstein von Artern* (ID: 1553) that was mentioned in a letter by J. G. Lenz in 1796, or the *Bernerde* (ID: 1563) Goethe bought the same year. Nevertheless, such pieces can provide insights into the time of acquisition. In the small amber collection, no handwritten label by Goethe exists, only empty form labels are within the small boxes.

***Supplementary results***

**†*Ctenobethylus goepperti* (Mayr, 1868).**

**=** †*Ctenobethylus succinalis* Brues, 1939: Brown 1977: 214.

**=** †*Eldermyrmex exsectus* Dubovikoff et Dlussky, 2019 (**syn. nov.**, **comb. nov.**).

**Protonym**: †*Hypoclinea goepperti* Mayr, 1868: p. 56; pl. 1, figs. 3–7; pl. 3, figs. 42–46.

Combination in *Bothriomyrmex*: Dalla Torre 1893: 170.

Combination in *Iridomyrmex*: Wheeler 1915: 90.

Combination in *Liometopum*: Shattuck 1992: 15.

Combination in †*Ctenobethylus*: Dlussky 1997: 58.

**Specimen data**. ID: 1552.b s 11 31 19. Scan ID: fsu_154_1552b_S11_31_19. Scan date: 23Nov-2022. Goethe amber collection (KSW, Klassik Stiftung Weimar).

**Description**.

***Morphometrics***. (N = 1, all metrics in mm, for abbreviations see material and methods section in the article) *Measurements*. A2L = 0.18; A3L = 0.12; CLL = 0.25; EL = 0.18; EW = 0.12; FCS = 0.29; HL1 = 0.88; HL2 = 0.91; HLA = 0.25; HLP = 0.41; HW = 0.91; IOC = 0.59; LOC = 0.03; ML = 1.04; PL = 0.25; SL = 0.58; SPD = 0.04. *Indices*. AI = 62.7; CI = 104; CS = 0.89; EI =152; ES = 19.8; OI = 96.4; SI = 63.7.

***Head***. The head is cordate, with posteriorly bulging occipital lobes; it is longer than broad. The clypeus extends posterad between the antennal toruli; its anterior margin is more-or-less straight, without an anteromedian notch or lobe and without paramedial emarginations or shoulders. The cranial condyles (= anterior mandibular articulations) are large. The frontal carinae are wide-set and well-defined, with sharp margins; they diverge posteriorly. The compound eyes are situated in the anterior half of the head and are distinctly separated from the lateral head margins; each eye is comprised of > 50 ommatidia. The medial hypostomal lamella is present, *i.e.*, the medial hypostoma is complete. The hypostomal teeth are scarcely developed.

*Appendages*. The scapes are shorter than both the head length and width; they would not exceed the posterior head margin if they were in repose; they are distinctly curved and thicken apically. The pedicel is elongate, being at least twice as long as wide. The third antennomere (= first flagellomere) is short, being slightly more than half the length of the pedicel. The flagellum is shorter than the mesosoma. The mandibles are robust, with over 10 teeth, which are uniseriate and continue onto the clearly differentiated basal margin; at rest, the mandibles are largely exposed. The palp formula is 6, 4. The maxillary palps are short, not reaching the postocciput and exceeding the hypostomal margin by somewhat more than the apical three palpomeres. The labial palps are short, not reaching the hypostomal margin. The maxillary stipes are smooth externally, without ridges or carinae on their external surfaces. The prementum is smooth and does not appear to have furrows defined by carinae. The labrum is lateromedially narrow, being not much wider than the medial hypostoma, and is deeply notched apically.

*Tentorium* (Fig. 4a). (*Note*: Character and state numbers from Richter et al. 2022, “REA”, indicated in parentheses.) The anterior tentorial arms are apparently weakly sinuous along their length and are not connected to the ventral surface of the head capsule (REA: char. 52, state 0). The medial tentorial lamellae are present (REA: char. 48, state 1); they are oriented parallel to the anterior tentorial arm (REA: char. 49, state 0); they are long, extending anteriorly almost to the anterior tentorial pit and posteriorly almost to the tentorial bridge (REA: char. 50, state 1); they are mostly flat (REA: char. 51, state 0), and are narrow along their lengths, apparently widest at about its midlength. The lateral tentorial lamellae are present (REA: char. 53, state 1); they are free from the head capsule (REA: char. 54, state 0); they are about half the width of the medial lamella and slightly broader; and they reach the dorsal tentorial arm but not the tentorial bridge (REA: char. 55, state 0). The dorsal tentorial arms apparently present (REA: char. 56, state 0) and short, being ≤ 1/3 the length of the anterior tentorial arms (REA: char. 57, state 0). The tentorial bridge is weakly convex, nearly linear (REA: char. 61, state 0), and not curving above the anterior arms (REA: char. 60, state 0); occurrence of a ventral ridge on the bridge is uncertain (REA: char. 62, state?). The anteromedial process of the tentorial bridge is present and apparently short. The posterior tentorial arms distinct but short (REA: char. 58, state 0); they are linear, and are aligned with the anterior tentorial arms, not bending ventrad and fusing to the head capsule (REA: char. 59, state 0).

***Mesosoma***. The mesosoma is compact; its length is about twice its height in lateral view. The pronotum does not have a defined anteromedian lobe; its anterior collar curves weakly to the convex posterior portion in lateral view; its posterior portion is relatively long and muscular in appearance. The propleurae are slightly longer than broad; they distinctly bulge ventrad the pronotum. The mesonotum is well-developed; it is about as long as the propodeum is tall; it is distinctly convex. The mesopleural region is distinguished from the mesonotum by curvature of the cuticle and bears an apparent epicnemial carina along its anterolateral surface. The ventral mesopectal region does not bear processes. The metanotum is distinctly developed in the focal specimen but may be poorly defined. The metanotal groove is impressed. The metapleural region is not visibly differentiated from the propodeum. The metapleural gland orifice is large and gaping, with the bulla distinctly bulging dorsad the orifice, and without associated carination. The ventral metapleural region does not have distinct processes. The propodeum is distinctly below the level of the mesonotum; it is short, with a dorsal surface that is distinctly less than half the dorsoventral height of the segment; its dorsal surface evenly and broadly curves into the posterior surface as seen in lateral view. The propodeal spiracle is situated at about propodeal midheight and is broad. The propodeal foramen is slightly produced posteriorly.

*Appendages*. The procoxa is about as twice as long as wide in lateral view. The profemur is thick relative to the protibia and is strongly convex ventrally. The strigil is weakly developed. Protarsomeres 2–4 are short, being as wide as long or only slightly longer than wide. The pretarsal claws of all legs are well-developed and apparently without teeth. The aroliae of all legs are distinct and well-developed. The mid and hind tibiae have one barbirulate spur each.

*Prosternum* (Fig. 4b). The basisternal area of the prosternum is in the form of a broad obtuse triangle as seen in an external, ventral view; the external surface of this area is flat, without apparent ridging, grooves, or pits. The posteromedial process (= “furcasternal process”) is apparently not developed, rather the basisternal and furcasternal areas curve evenly into one another. In lateral internal view, the basisternal and furcasternal regions of the prosternum are nearly perpendicularly oriented. Internally, the prodiscrimen is either poorly developed or poorly preserved; in either case, it ends posteriorly well ventrad the furcal arms. The furcasternal region is subrectangular in anterior view, being slightly taller dorsoventrally than wide lateromedially, with the lateral margins weakly converging toward the bases of the furcal arms; it is also flat, without the lateral surfaces converging posteromedially; and externally, it lacks a longitudinal ridge. The “horizontal plate” is not apparently developed. The furcal arms are short and narrow; they meet medially, thus forming the furcal bridge over the neural foramen. The dorsal furcal lamellae appear weakly developed and the ventral lamellae are scarcely developed.

***Metasoma***. The petiolar levator apodeme has a wide foramen. The anteroventral petiolar process (= subpetiolar process) is absent. The petiolar node is distinct and narrowly squamiform, rising above the levator apodeme dorsally. The posterior collar of the petiole is long but not grossly elongate. The ventral petiolar surface is convex but not produced as a strong lobe. The helcium is infraaxial; its tergite extends ventrad its sternite, which is weakly convex. The posttergites of abdominal segment III (= metasomal II) are tergosternally fused laterad the helcium; their articulation extends anterodorsally beyond the helcium becomes posteriorly directed through a narrow curve; due to this shouldering, the posttergites form a groove which receives the posterior collar of the petiole when the gaster is in repose. Four gastral tergites are visible in dorsal view, with the terminal tergum flexed ventrad. The terminal sternite is weakly keeled longitudinally.

**Comments.** We synonymize †*Eldermyrmex exsectus* Dubovikoff & Dlussky, 2019 under †*Ctenobethylus goepperti* (Mayr, 1868) **syn. nov.**, resulting in the combination †*Ctenobethylus exsectus* (**comb. nov.**). Based on the photomicrographs of †*C. exsectus* provided in the original publication, most of the 11 diagnostic conditions of †*Ctenobethylus* provided above can be confirmed, with the exception of the short pedicel, as the flagellae are missing, and the exact form of the petiole, which is imperfectly preserved. Specifically, †*C. exsectus* has a cordate head, large and robust mandibles, short scapes, a compact mesosoma with a well-developed mesoscutum and a short propodeum, and the petiolar node appears to be developed and narrow squamate. The following considerations arise from the original publication: The CI of †*C. exsectus* is recorded as 120, which may be due to distortion of the specimen; the petiolar node is stated to be conical, but the exact form is difficult to ascertain; the holotype of †*C. exsectus* is smaller overall than the focal specimen in the present study, but has similar proportions, including ES (18 vs. 20) and SI (66 vs. 64). The primary justification for including †*C. exsectus* in †*Eldermyrmex* is the apparent propodeal ridging and petiolar node form of the poorly preserved type specimen. Presence of terminal sternite keeling is not of particular discriminatory value.

*Prosternum*. Presently, there is very little information available about the three-dimensional shape of the prosternum of ants. Indeed, structural knowledge of the prosternum is limited to *Formica* Linnaeus, 1758 and a single stem ant species. In comparison to *Formica*, the prosternum of †*Ctenobethylus* is differentiated by the apparent absence of the posteromedial prosternal process, the weak development of the discrimenal lamella and dorsal furcal lamellae, the proportionally longer furcasternal region as seen in anterior view, the flatness of the furcasternal region externally and internally, and the more wideset furcal arms, resulting in a proportionally larger neural foramen.

*Tentorium*. Among the taxa for which tentorial anatomy has been rendered from µ-CT scans (†*Gerontoformica*, *Protanilla*, *Brachyponera*, *Tatuidris*, *Dorylus*, *Formica*, *Wasmannia*), the tentorium of †*Ctenobethylus* is most similar to *Formica*. Specifically: (1) the anterior arms aligned with the posterior arms (also occurring in †*Gerontoformica*, *Protanilla*, and *Wasmannia*, vs. posterior arms curved ventrad in *Tatuidris*, curved dorsad in *Brachyponera*, virtually absent in *Dorylus*); (2) the medial lamella is long and narrow (also observed in *Brachyponera* and *Wasmannia*, although the lamella does not extend as far posterad toward the tentorial bridge in the latter). The lateral lamella differs from *Formica* and *Brachyponera* in being short and wide, while this lamella is poorly developed to absent in *Protanilla*, absent in *Tatuidris*, *Dorylus*, and *Wasmannia*, and uncertain in †*Gerontoformica*.

***Supplementary figures***


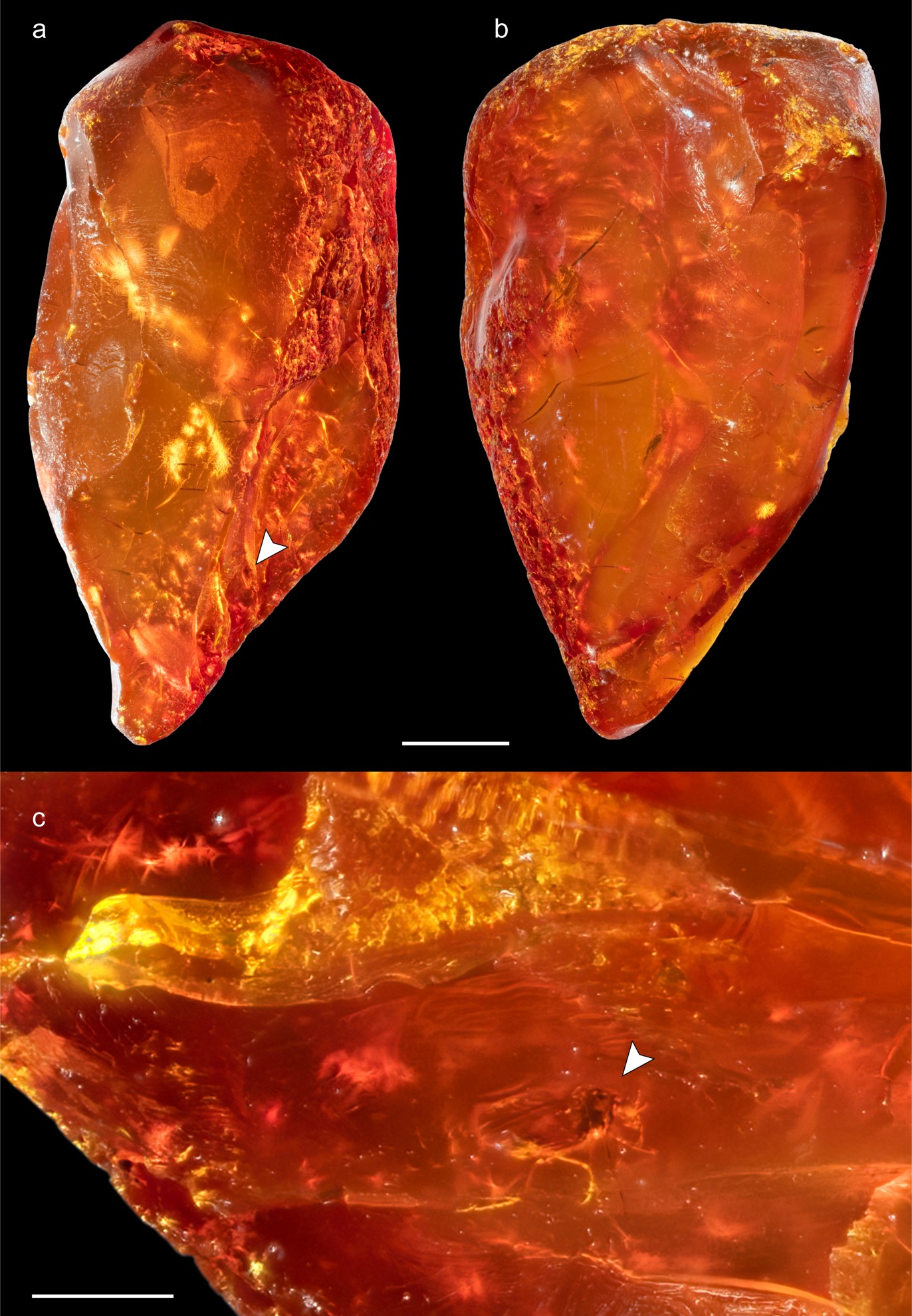


**Fig. 1 | Amber piece 1550.d two side view.** **a** Visible inclusion of a Simuliidae. **b** backside of the amber piece. **c** Close up of the Simuliidae. Arrows indicate the Simuliidae inclusion. Scale bar 5 mm (top) and 1 mm (bottom).


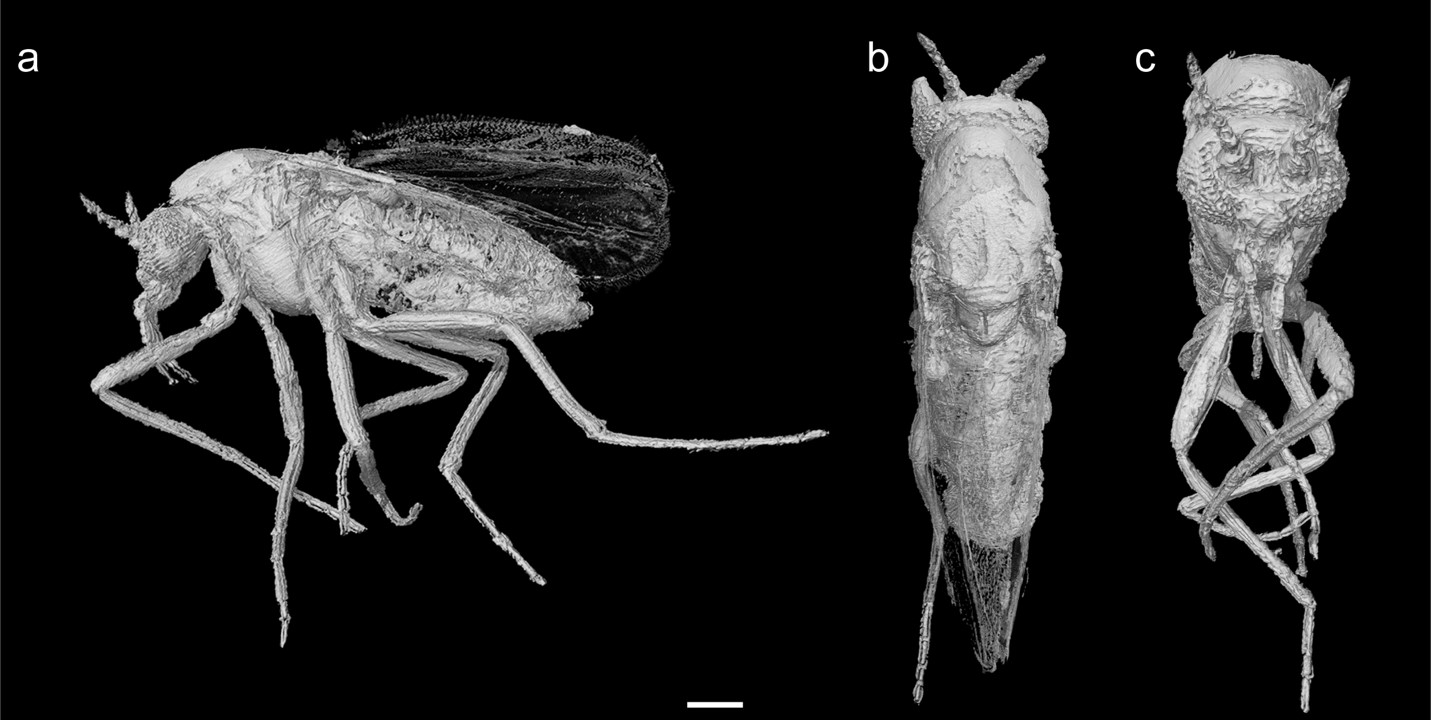


**Fig. 2 | 3D renders of blackfly (Simuliidae) in amber piece 1550.d.** **a** Lateral view. **b** Dorsal view. **c** Frontal view. Scale bar 100 µm. 3D model available on Sketchfab: [https://sketchfab.com/3dmodels/simuliidae-a95ab4aa04924e6ea1ac3d4a76eaf870.](https://sketchfab.com/3d-models/simuliidae-a95ab4aa04924e6ea1ac3d4a76eaf870)


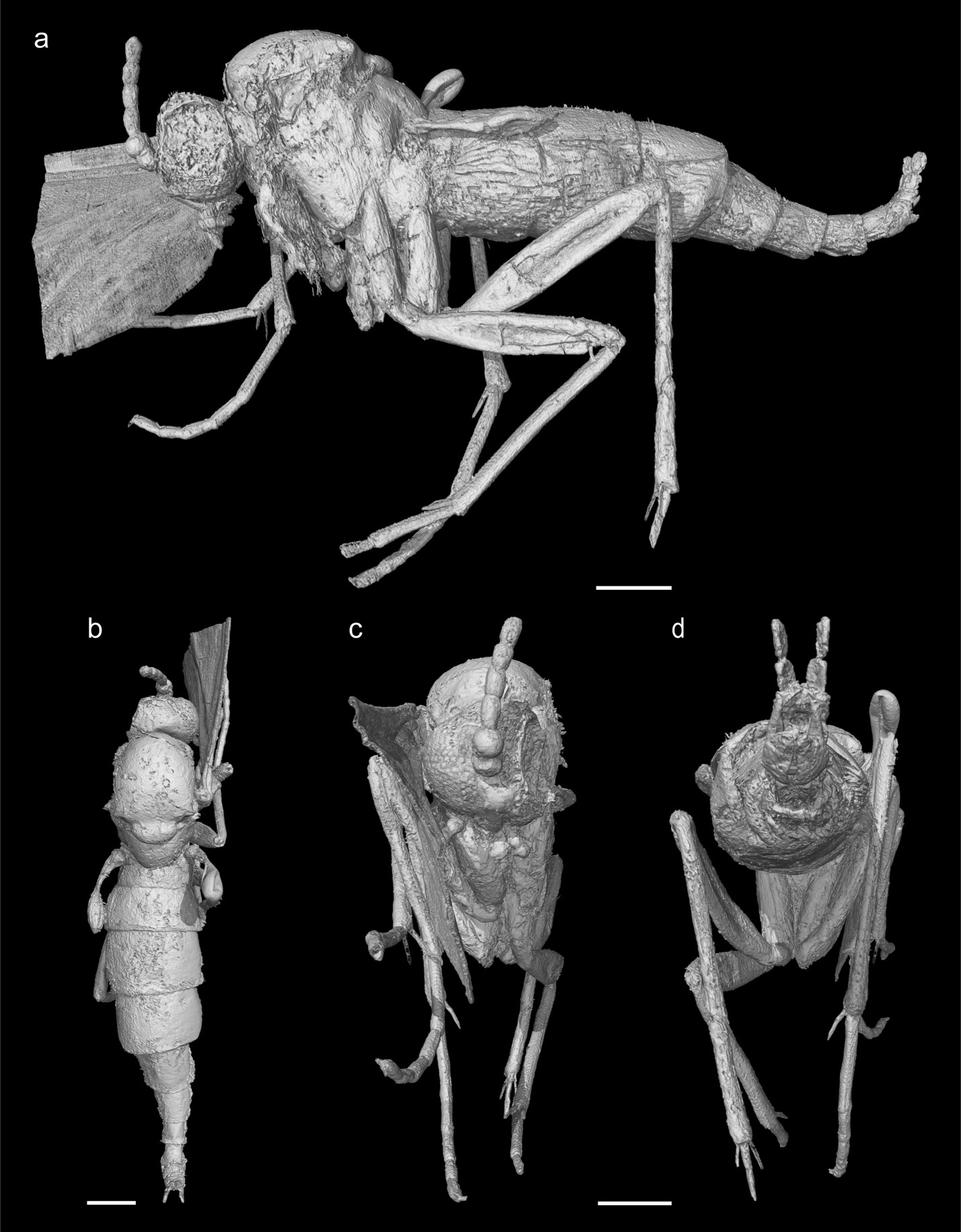


**Fig. 3 | 3D renders of dark-winged fungus gnat (Sciaridae) in amber piece 1552.b**. **a** Lateral view. **b** Dorsal view. **c** Frontal view. **d** Caudal view. Scale bars 250 µm. 3D model available on Sketchfab: [https://sketchfab.com/3d-models/sciaridae-0192c50b698644beb7952a2e68f74d6b.](https://sketchfab.com/3d-models/sciaridae-0192c50b698644beb7952a2e68f74d6b)

***Supplementary text box***

| Foreword to the inaugural issue of *Nature* (Huxley 1869):    THURSDAY, NOVEMBER 4, 1869 *NATURE: APHORISMS BY GOETHE.*    ‘NATURE! We are surrounded and embraced by her: powerless to separate ourselves from her, and powerless to penetrate beyond her. … If we consider the high achievements by which all the phenomena of Nature have been gradually linked together in the human mind… we shall, not without a smile… rejoice in the progress of fifty years.’ (essay continues) (Goethe 1828)  …  It seemed to me that no more fitting preface could be put before a Journal, which aims to mirror the progress of that fashioning by Nature of a picture of herself, in the mind of man, which we call the progress of Science.  Forty years have passed since these words were written. [Goethe’s] notions… are now the commonplaces of science… When another half-century has passed, curious readers of the back numbers of *Nature* will probably look on *our* best, ‘not without a smile;’ and, it may be, that long after the theories of the philosophers whose achievements are recorded in these pages, are obsolete, the vision of the poet will remain as a truthful and efficient symbol of the wonder and the mystery of Nature. |
| --- |

Even though today the authorship of Nature is more likely to be attributed to Georg Christoph Tobler (1757 –1812) than Goethe, a fact that maybe Huxley was already aware of, as he wrote “*But when that original is Goethe’s […]*” (p. 10). Nevertheless, to still choose it as an opening article for Nature shows the high regard Huxley had for Goethe, as well as the influence Goethe had on natural science.
